# The gender and geography of publishing: a review of sex/gender reporting and author representation in leading general medical and global health journals

**DOI:** 10.1136/bmjgh-2021-005672

**Published:** 2021-05-13

**Authors:** Rebekah Merriman, Ilaria Galizia, Sonja Tanaka, Ashley Sheffel, Kent Buse, Sarah Hawkes

**Affiliations:** 1 Global Health 50/50, London, UK; 2 C&H Mental Health Medical Adult, East London NHS Foundation Trust, London, UK; 3 Department of International Health, Johns Hopkins Bloomberg School of Public Health Center for Teaching and Learning, Baltimore, Maryland, USA; 4 Director, Healthier Societies Program, the George Institute for Global Health, Newtown, New South Wales, Australia; 5 Institute for Global Health, University College London, London, UK

**Keywords:** descriptive study, public health

## Abstract

**Introduction:**

Diverse gender and geographical representation matters in research. We aimed to review medical and global health journals’ sex/gender reporting, and the gender and geography of authorship.

**Methods:**

542 research and non-research articles from 14 selected journals were reviewed using a retrospective survey design. Paper screening and systematic data extraction was conducted with descriptive statistics and regression analyses calculated from the coded data. Outcome measures were journal characteristics, the extent to which published articles met sex/gender reporting guidelines, plus author gender and location of their affiliated institution.

**Results:**

Five of the fourteen journals explicitly encourage sex/gender analysis in their author instructions, but this did not lead to increased sex/gender reporting beyond the gender of study participants (OR=3.69; p=0.000 (CI 1.79 to 7.60)). Just over half of research articles presented some level of sex/gender analysis, while 40% mentioned sex/gender in their discussion. Articles with women first and last authors were 2.4 times more likely to discuss sex/gender than articles with men in those positions (p=0.035 (CI 1.062 to 5.348)). First and last authors from high-income countries (HICs) were 19 times as prevalent as authors from low-income countries; and women from low-income and middle-income countries were at a disadvantage in terms of the impact factor of the journals they published in.

**Conclusion:**

Global health and medical research fails to consistently apply a sex/gender lens and remains largely the preserve of authors in HIC. Collaborative partnerships and funding support are needed to promote gender-sensitive research and dismantle historical power dynamics in authorship.

**Key questions**

**What is already known?**

- Sex and gender as determinants of health are overlooked in research and literature. Data derived from male participants have long been considered the medical norm and has resulted in an evidence base that fails to be fully replicable across genders.
- A growing body of research suggests an association between the gender of the researchers and the consideration and application of sex/gender analysis in their research.
- Long-standing biases within medical research and academia favours male authors from high-income countries, resulting in women and those residing in low-income and middle-income countries (LMICs) having the least opportunity to publish.

**What are the new findings?**

- Despite global guidelines and editorial policies, integration of sex and gender analysis remains limited in health research across journal and article type.
- Both gender and geographical affiliation are separate but compounding determinants of authors’ access to publishing, and publishing in high impact journals, with women authors from LMICs particularly underrepresented.
- Women in senior authorship positions are more likely to produce research articles that have more women in total authorship, as well as discuss sex/gender differences.

**Key questions**

**What do the new findings imply?**

- Inadequate sex/gender analysis within research results in missed opportunities for gender-responsive policies, programmes and interventions. Clear, consistent and enforceable guidance is needed that covers the spectrum of biomedical and social science research.
- The significant under-representation of LMIC authors in global health publishing needs to be urgently addressed to allow for the production of context-sensitive and culturally appropriate research.

## Introduction

Sex and gender are both important determinants of health.^1^ Sex generally refers to the biological characteristics associated with being identified as male, female or intersex, while gender, as a social construct, encompasses both patterns of social organisation as well as ascribed roles, positions and expected behaviours for all people in all societies.^2^ Individually and together, sex and gender exert an influence on a person’s health and well-being across the life-course.^3^ The importance of accounting for the role of sex and gender in medical and health policy, practice and research is well-established.^4–8^ In their recent review of sex and gender as modifiers of health, disease and medicine, Mauvais-Jarvis *et al* highlighted the fundamental role that sex and gender play in determining both morbidity and mortality through the intersection of biological characteristics and social position.^1^ The authors urge researchers to consider sex and gender both ‘throughout the research process’ and in the practice of medicine as ‘essential for the success of clinical care and translational science’. The COVID-19 pandemic, for example, has highlighted the role that sex-related differences in immune function are playing in determining susceptibility and response to viral infection, while gender is thought to play an important role in driving the lower rates of testing uptake seen in men compared with women.^9 10^ This may be contributing to the recorded mortality differences through women’s lower rates of death registration compared with men.

Within academic publishing, guidelines exist for systematic reporting and analysis of sex and gender in research. The International Committee of Medical Journal Editors (ICMJE) Recommendations, as the primary source of author guidance for manuscript preparation in the biomedical sciences, encourage researchers to ‘aim for inclusive representative populations in all study types’ for ‘such variables as age, sex, or ethnicity’.^11^ The Sex and Gender Equity in Research (SAGER) guidelines, published in 2016, outline the importance and benefits of taking sex and gender into account in medical and health research, including across the entire publishing process—from submission, through peer review and publication. These guidelines were endorsed by the Committee on Publication Ethics in 2018, which provides best practice and guidance to editors and publishers.^12^

Systematically pursuing a clearer understanding of the influence and intersection of sex and gender on health and health equity would improve the depth and targeting of medical research, allow more informed and equitable decisions in health policy, and ultimately contribute to better health outcomes for all.^2 13^ However, despite stringent guidelines and widespread advocacy, sex and gender remain consistently overlooked or conflated in medical and health research—from study design to implementation and reporting.^2 14^ The absence of consideration of sex and gender produces research that fails to address either biological or socially constructed differences between people, resulting in lost opportunities for effective policies, programmes, practices and interventions.

Gender inequalities also affect the career opportunities of individuals involved in research and publishing. Long-standing biases within research organisations, funding bodies, and academia favour male authors and editors, often situated in institutions in high-income countries (HICs).^15–17^ Women continue to be under-represented among authors, particularly in senior positions and higher impact journals.^18 19^ Gender inequality in publishing has been highlighted again during the COVID-19 pandemic, where women represent just one-third of authors publishing on the topic,^20^ a figure similar to previous findings.^21 22^ Economy and location also determine authorship and opportunity; multiple studies demonstrate the under-representation of authors from low-income and middle-income countries (LMICs).^23 24^ A lesser explored area is how geography intersects with gender in authorship, with only a few studies demonstrating that women from LMICs are particularly under-represented.^25 26^ Again, there are some efforts and initiatives to redress these imbalances. National governments and funding agencies, for example, have implemented initiatives to promote gender equality in careers in science.^27–29^ Additionally, universities and other research organisations participate in a variety of initiatives to promote gender equality—ranging from systems of external grading through charter marks, to mandatory training programmes designed to address bias in the workplace.^30^ Some journals, such as *The Lancet* group and *BMJ Global Health*, have also made important pledges and progress towards more equitable publishing processes.^31 32^

Nonetheless, despite commitments and concerns across these two interrelated domains—that is, the importance of sex and gender analysis in published research, and ensuring equity in the realm of research, authorship and publishing—evidence on the extent to which these two objectives are intertwined in health and medical publishing is sparse. We, therefore, undertook a review of these two domains across 14 journals publishing in the general medical/health fields and the specialist field of global health. The objectives of the research were to analyse: (1) the extent to which the articles published abide by guidelines on reporting sex and gender; (2) the gender and regional affiliation of the authors publishing in these journals; and (3) the relationship between these author characteristics and reporting on sex and gender in published work.

## Methods

We purposively selected 14 general medical and specialist global health journals according to their impact in the field of medical publishing (general journals, n=6) or their specific focus on global health (n=8) (see box 1). All 14 journals aim to inform and influence international health scholarship, either through their reach (all six general medical journals reference their international readership on their websites), or through their primary focus on publishing content related to global health.

Box 1 **Journals included in our sample**

**General medical journals (n=6)**

*BMJ*

*JAMA*

*JAMA Internal Medicine*

*Lancet*

*New England Journal of Medicine*

*PLOS Medicine*

**Specialist global health journals (n=8)**

*BMC Globalisation and Health*

*BMJ Global Health*

*Global Health Action*

*International Journal of Health, Policy and Management*

*Journal of Global Health*

*Journal of Health, Population and Nutrition*

*Lancet Global Health*

*WHO Bulletin*

**Figure 1:**
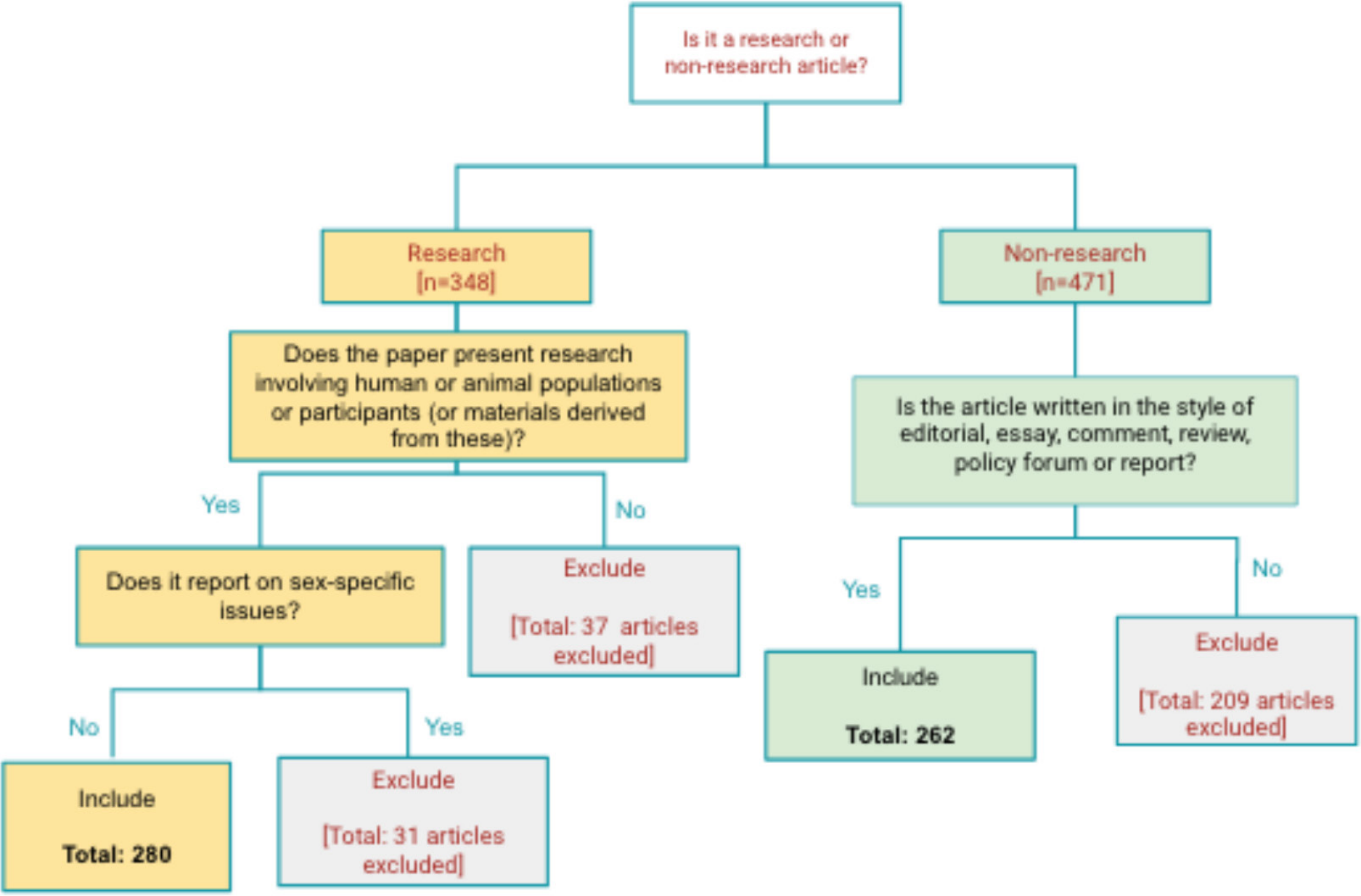
Article inclusion/exclusion process. For each article published in each of the 14 journals under review, starting from 1st January 2018, the following inclusion/exclusion process was applied.

For each journal, we identified and assessed the first 20 research articles and the first 20 non-research articles (editorials, commentaries, reviews, etc.) published in issues starting on 1 January 2018 (see figure 1). This sampling frame was selected to provide a standardised means of assessing publications across multiple journals within a similar timeframe. A total of 280 research articles and 262 non-research articles were reviewed (one journal published only two non-research articles during the timeframe under review from 1 January 2018−1 June 2019). Articles in supplement issues were excluded.

Research articles were defined as being any article publishing original or new material, including primary and secondary research. Articles reporting research (including systematic reviews of research) included human or animal populations or participants or material derived from these. Studies reporting on sex-specific issues (eg, cervical or prostate cancer) were noted but excluded from additional analyses. This was to avoid bias from studies only able to include one sex, which would have automatically skewed the results of our analysis using the assessment criteria outlined. Non-research articles included reviews, reports, policy forums, viewpoints, commentaries and editorials. Journal-specific article types, methodologies, case reports, obituaries, news, letters, book reviews, or images and other media were excluded.

### Journal characteristics

The journal impact factor^33^ for each journal assigned by the Clarivate Analytics Journal Citations Reports was extracted for 12 of the 14 journals under review. Two of the journals did not have a Clarivate Analytics journal impact factor (*BMJ Global Health* and *WHO Bulletin*). The impact factors for these two journals were extracted from their individual websites. The author guidelines of each journal were reviewed to determine whether and in what context they referenced ICMJE and/or SAGER guidelines.

### Article review

Two reviewers independently examined each article and evaluated the extent to which sex/gender was accounted for in its research and reporting, as well as the gender, geographic affiliation and nationalities of its authors. For research articles, assessment was aligned with the criteria set out in the SAGER guidelines, which we used as a proxy for the extent to which sex and gender related reporting was done (box 2). For non-research articles, a simple assessment was made as to whether sex/gender was the main topic of the piece or mentioned as part of the broader text (box 3). Disagreements between reviewers were resolved by consensus: any remaining discrepancies were adjudicated by a third reviewer. Boxes 2 and 3 present the frameworks applied in reviewing research and non-research articles.

Box 2 **Review framework for assessing research articles’ alignment with Sex and Gender Equity in Research guideline criteria**

**Pre-specification**

**Does the title, abstract or introduction set out an aim to analyse, discuss or disaggregate sex/gender?**

*Example text: “This paper examines mobile phone usage for obtaining health information in Mirzapur, Bangladesh. It contrasts college students’ usage with that of the general population, asks whether students are using digital technologies for health information in innovative ways, and examines how gender affects this.” (Waldman, BMC Globalisation and Health*)

*Assessment: yes*

**Gender of participants**

**Does the article state the proportion of male/female participants? If yes, what is the percentage of female participants?**

*Example text: “The final study cohort consisted of 405 878 participants (48.0% men and 52.0% women).” (Tu, BMJ*)

*Assessment: yes*

For articles containing 0%–25% or 75%–100% female participants, further details were noted as follows:

**If only one sex and/or gender is included, does the title specify the sex/gender of participants?**

*Example text: “Social-ecological factors associated with selling sex among men who have sex with men in Jamaica: results from a cross-sectional tablet-based survey” (Logie, Global Health Action*)

*Assessment: yes*

**If women (or men) represent less than 25% of participants, does the article make any attempt to explain, justify or discuss as a limitation?**

*Example text: “Study limitations include the retrospective study design and potential biases in the sample, which may overrepresent men with symptomatic infection…” (Yin, PLOS*)

*Assessment: yes*

**Determining sex/gender**

**Does the article state how sex and/or gender of study participants was determined for example, through self-reporting, genetic testing or any other means?**

*Example text: “Participants were interviewed and asked for self-reported information regarding age, gender,…” (Pokhrel, GHA*)

*Assessment: yes*

**Analysis of sex/gender**

Does the article consider, analyse or discuss any differences in results between male and female participants?

*For randomised control trials (RCTs), further detail was obtained:*

**Does the article consider or discuss any differences in findings between male and female participants?**

*Example text: “As is common in the black South African population, obesity was more prevalent in women than men.’ (Goudge, BMJ GH*)

*Assessment: yes*

**Does the article factor in or adjust its analysis by sex?**

*Example text: “The model was also adjusted for age, sex… ” (Huffman, JAMA*)

*Assessment: yes*

**Does the article apply any statistical model or analysis to its findings to differentiate between male/female participant outcomes?**

*
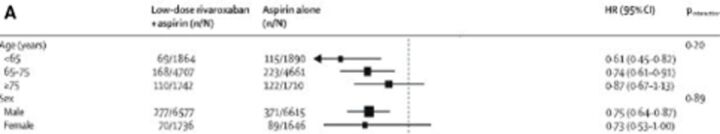
*

*(Connely, Lancet)*

*Assessment: yes*

**Discussion of sex/gender**

**Does the article make reference to sex and/or gender (either relating to the study’s findings or in a more general sense) in the discussion section of the article?**

*Example text: “We found that households headed by females were at a higher risk of catastrophic health expenditure, indicating that there are gender differences in the capacity to pay for healthcare.” (Pandey, WHO*)

*Assessment: yes*

Box 3 **Review framework: Non-Research articles**

**Sex/gender topic**

**Is the title or main theme of the article on a topic specifically relating to issues of sex and/or gender?**

Example text: *“Involving both parents in HIV prevention during pregnancy and breastfeeding” (Chi, WHO*)

*Assessment: yes*

**Does the article mention sex and/or gender?**

*Example text: “the effectiveness of this communication was undermined by the broader social and gender hierarchies that limited participation in these councils” (Mbau, GHA*)

*Assessment: yes*

### Author characteristics: determining gender and institutional affiliation of authors

The gender of each author for all research and non-research articles was determined by a two-step process. First, by inputting the first name on genderchecker.com. Second, by manual internet search to confirm gender via pronouns used in online biosketches. Individuals were coded as male, female, non-binary or unknown (if unable to verify).

Data on country of institutional affiliation were collected for first and last authors. The affiliated institution provided in the article was used and location confirmed via internet search. Countries were then assigned to their World Bank Classification of economic category (high, middle or low income).^34^

### Statistical analysis

Descriptive statistics were used to summarise the extent of sex/gender reporting overall, in general medical journals and in specialist global health journals, gender of authors, and country of author institutional affiliation. For all gender and geographical affiliation analyses, where the gender or geographical affiliation of authors could not be determined, these authors were excluded from the analysis - resulting in the exclusion of 20 authors. All single author articles (65 articles, 62 of which were non-research articles) were included only in the analysis of first author data. In addition, for non-research articles, articles authored by editors of journals were excluded from the gender/geographical affiliation analysis (n=11).

Bivariate logistic regression analysis was performed to assess the relationship between reporting on sex and gender in published work and author characteristics. Bivariate linear regression analysis was performed to assess the relationship between journal impact factor and author characteristics. Author characteristics included first and last author gender composition, first and last author gender pairing, first author geographic affiliation, and last author geographic affiliation. Regression analyses excluded single author articles. All analyses were completed in STATA V.15 (StataCorp).

## Findings

### Journal instructions to authors

At the time of writing, 5 (2 general medical, and 3 global health) out of the 14 journals under review explicitly encourage sex and gender analysis and the correct use of sex/gender terms in their instructions to authors (table 1). Additionally, five journals (four general medical, one global health) also promote attention to factors other than sex/gender, most notably race/ethnicity and age.

**Table 1:** Journals under review: impact factor, reference to International Committee of Medical Journal Editors (ICMJE) and Sex and Gender Equity in Research (SAGER) or sex/gender related reporting in author instructions

	General Medical (GM) or Global Health (GH) Journal	Impact factor (2018)	Reference ICMJE?	Reference SAGER?	Author instructions reference sex/gender	Author instructions reference other characteristics for example, race, age
*BMC Globalisation and Health*	GH	2.554	Yes	No	No	No
*BMJ*	GM	27.604	Yes	No	No	Yes
*BMJ Global Health*	GH	4.280	Yes	Yes	Yes	No
*Global Health Action*	GH	1.817	Yes	No	No	No
*International Journal of Health, Policy and Management*	GH	4.485	Yes	No	No	No
*JAMA*	GM	51.273	Yes	No	Yes	Yes
*JAMA Internal Medicine*	GM	20.768	Yes	No	Yes	Yes
*Journal of Global Health*	GH	3.079	Yes	No	No	No
*Journal of Health, Population and Nutrition*	GH	1.828	Yes	No	No	No
*Lancet*	GM	59.102	Yes	No	Yes	Yes
*Lancet Global Health*	GH	15.873	Yes	No	Yes	Yes
*New England Journal of Medicine*	GM	70.670	Yes	No	No	No
*PLOS Medicine*	GM	11.048	Yes	No	No	No
*WHO Bulletin*	GH	6.818	Yes	No	No	No

All journals reference ICMJE, but generally only in the context of the accuracy and integrity of submissions and policies around competing interests disclosures, not with regards to its sex/gender reporting guidance. Only one journal, *BMJ Global Health*, currently references the SAGER guidelines in its instructions for authors.

Research articles from journals that specified sex/gender reporting in their author guidelines were 3.69 times more likely to report the sex/gender of study participants, compared with articles in journals that do not (OR: 3.69, p=0.000 (CI 1.79 to 7.60)). However, no further correlation was found between these journals and other areas of sex/gender reporting (online supplemental table 1).

10.1136/bmjgh-2021-005672.supp1 Supplementary data

### Extent to which published articles include sex/gender related reporting and analysis

#### Research articles

Among the 280 research articles included in our sample, 23 (8%) prespecified the intention to undertake an analysis of sex/gender (table 2). The sex/gender of study participants was reported in 201/280 (72%) of articles, with 2/280 (0.7%) reporting on proportions of transgender or non-binary participants. Among the articles reporting on the sex/gender of study participants, women on average represented half of all participants. However, among the 24 articles that had a majority (over 75%) of 1 sex/gender, in 19 (80%) there was no reporting explaining the imbalance.

**Table 2:** Comparing sex/gender reporting in general medical journals and global health specialist journals, by research and non-research articles

Sex/gender reporting framework	Research articles			Non-Research articles		
	Total (n=280)	General medical journals (n=120)	Global health specialist journals (n=160)	Total (n=262)	General medical journals (n=120)	Global health specialist journals (n=142)
Pre-specified sex/gender analysis	8%	6%	10%			
Reported gender of study participants	72%	91%	57%			
Percentage of female participants						
0%–25%	8%	8%	8%			
25%–50%	43%	47%	36%			
50%–75%	43%	41%	45%			
75%–100%	7%	4%	11%			
Reported participation of transgender/non-binary participants	0.7%	0%	1%			
Stated how sex/gender was determined	6%	5%	6%			
Performed any form of analysis of sex/gender differences (excluding eight articles w/ single sex participants)	59%	71%	50%			
Featured sex and/or gender in discussion	40%	29%	49%			
Focused on an issue specifically relating to sex/gender				8%	6%	10%
Referred to sex/gender related issues anywhere in text				40%	29%	50%

The dark grey colour indicates there is no data in that cell.

The number of articles that defined how the sex/gender of study participants was determined was 16/280 (6%).

Fifty-nine per cent (161/272) of articles performed some form of analysis—including statistical and narrative—of sex/gender differences. Forty per cent (113/280; 40%) of articles applied a sex/gender lens in discussing their findings. This includes those articles that explored how sex/gender may have had an impact on reported results, as well as those that discussed their lack of a sex/gender analysis as a limitation.

A larger proportion of research articles published in general medical journals reported the sex/gender of participants than those published in specialist global health journals (91% vs 57%). Similarly, a larger proportion of articles published in general medical journals undertook some form of sex/gender analysis (71% vs 50%) than those in specialist global health journals. However, articles published in specialist global health journals were more likely to reference issues of sex/gender in their discussion sections than those in general medical journals.

Randomised control trials (RCTs) represent the gold standard of scientific research and therefore are subject to rigorous reporting standards. Among the 44 RCT studies included in our sample, 93% (41/44) reported the sex/gender of participants and two-thirds (29/44; 66%) performed any kind of analysis of sex/gender differences, including reporting results for male and female participants separately. However, robust analysis of sex and gender was notably missing from RCTs, with just 18% of articles performing more than one type of analysis (see box 2). One-quarter of RCTs (12/44; 27%) then went on to mention sex/gender in the discussion of their findings (online supplemental table 2).

10.1136/bmjgh-2021-005672.supp2 Supplementary data

Qualitative research methods represent another important approach to knowledge production. In the small sample of 33 qualitative articles in our study, 55% (18/33) reported the sex/gender of their study participants, while just 24% (7/29) of those with both male and female participants performed any level of sex/gender analysis and 27% (9/33) referenced sex/gender in their discussion (online supplemental table 2).

#### Non-research articles

We reviewed 262 non-research articles for their consideration of sex/gender issues. We found that 8% (21/262) focused on an issue specifically relating to sex/gender and 40% (106/262) referred to sex/gender related issues anywhere in the text (table 2). Ten percent (14/142) of articles published in specialist global health journals focused on an issue related to sex/gender, compared with 6% (7/120) of articles in general medical journals. Half (50%; 71/142) of specialist global health journal articles discussed issues of sex/gender compared with 29% (35/120) of general medical journal articles.

### Gender and geographical affiliation of authors

#### Author gender

Among all research article authors whose gender could be identified, 36% (1013/2778) were women. In the same sample, 40% (107/270) of first authors were women, and 23% (63/272) of last authors were women. Overall, four times as many articles had male first and last authors as female first and last authors (table 3).

**Table 3:** Representation of women in authorship, by journal type and article type

	General medical journal—research	Specialist Global Health journal— research	General medical journal— non-research	Specialist Global Health journal— non-research
**Women**				
First authors	32% (38/117)	45% (69/153)	31% (34/111)	52% (72/138)
Of whom are from LMICs	3% (1/38)	29% (20/69)	3% (1/33)	19% (14/72)
Last authors	17% (20/119)	28% (43/153)	21% (15/73)	40% (46/115)
Of whom are from LMICs	10% (2/20)	28% (12/43)	7% (1/15)	20% (9/46)
Single authors	–	–	21% (8/38)	35% (8/23)
Of whom are from LMICs			14% (1/7)	0% (0/8)
**Men**				
First authors	68% (79/117)	55% (84/153)	69% (77/111)	48% (66/138)
Of whom are from LMICs	6% (5/79)	43% (36/84)	4% (3/77)	29% (19/66)
Last authors	83% (99/119)	72% (110/153)	79% (58/73)	60% (69/115)
Of whom are from LMICs	2% (2/99)	34% (37/110)	2% (1/58)	25% (17/69)
Ratio of articles with first and last women authors vs first and last men authors	1:8	1:3	1:5	1:1

LMICs, low-income and middle-income countries.

Women-led research articles were more likely to have more women in total authorship than men-led articles: articles with women last authors had on average 54% women coauthors, while those with male last authors had 34% women co-authors.

Across the non-research articles, 42% (406/974) of all identifiable authors were women. Among articles with multiple authors, 32% (61/188) had women as last authors, while 26% (16/61) of single-author articles were by women. Non-research articles with women last authors had on average 55% women coauthors, while those with men last authors had 41% women coauthors (table 3). Additionally, for both research and non-research articles, global health specialist journals had higher proportions of both women first and last authors than general medical journals.

Among all women authors, 71% (1013/1419) published research articles and 29% (406/1419) published non-research articles. For men authors the corresponding figures are 76% (1765/2333) and 24% (568/2333). Overall, men were more likely than women to publish research articles, which is statistically significant (p=0.004).

#### Relationship between author characteristics and reporting on sex and gender

We explored associations between the sex/gender variables used to analyse the articles, and the gender of first and last authors. For research articles, women-led articles were more likely to discuss sex/gender. Articles with a woman first and last author were 2.4 times as likely to reference sex/gender in the discussion of their findings, as compared with articles with men first and last authors (p=0.035 (CI 1.062 to 5.348)). Articles with women first and last authors were also 2.2 times as likely to reference sex/gender in the discussion of their findings compared with other author gender pairings (p=0.039 (CI 1.0425 to 4.858)). We did not find any notable significant associations with the other four SAGER variables (table 4).

**Table 4:** ORs from logistic regression of author gender on reporting on sex and gender†

	Prespecified sex/gender analysis	Reported gender of study participants	Stated how sex/gender was determined	Performed any form of sex/gender analysis	Sex or gender in discussion
**Research articles**					
Author composition (first, Last)					
FF	REF	REF	REF	REF	REF
MF	0.33	0.63	1.93	0.40	0.46
FM	0.28	0.76	0.79	0.56	0.48
MM	0.43	1.66	2.48	1.05	0.42*
Author gender pairing (First and last author female)					
MF/FM/MM	REF	REF	REF	REF	REF
FF	2.73	0.92	0.54	1.33	2.25*
Author gender pairing (First or last author female					
MM	REF	REF	REF	REF	REF
FM/MF/FF	1.05	0.47*	0.45	0.57*	1.39
**Non-research articles**					
	**Focused on an issue specific to sex/gender**	**Referred to sex/gender anywhere in text**			
Author composition (first, Last)					
FF	REF	REF			
MF	0.26	0.31*			
FM	0.37	0.71			
MM	0.56	0.56			
Author gender pairing (First and last author female)					
MF/FM/MM	REF	REF			
FF	2.27	1.79			
Author gender pairing (first or last author female					
MM	REF	REF			
FM/MF/FF	0.95	1.18			

*p<0.05.

†Table presenting the likelihood that an article with different gender pairings for first and last authors have met the five variables assessed. <1 = less likely and >1 = more likely. The gender pairing against which the others are compared is indicated by REF. Significant associations are indicated by an asterisk.

Additionally, for non-research articles, we did not find any notable significant associations (table 4). While not statistically significant, we found that articles in our sample with first and last women authors were 1.79 times more likely to reference sex/gender than articles with first and/or last male authors.

#### Author institutional affiliation: region and country income level

**Figure 2:**
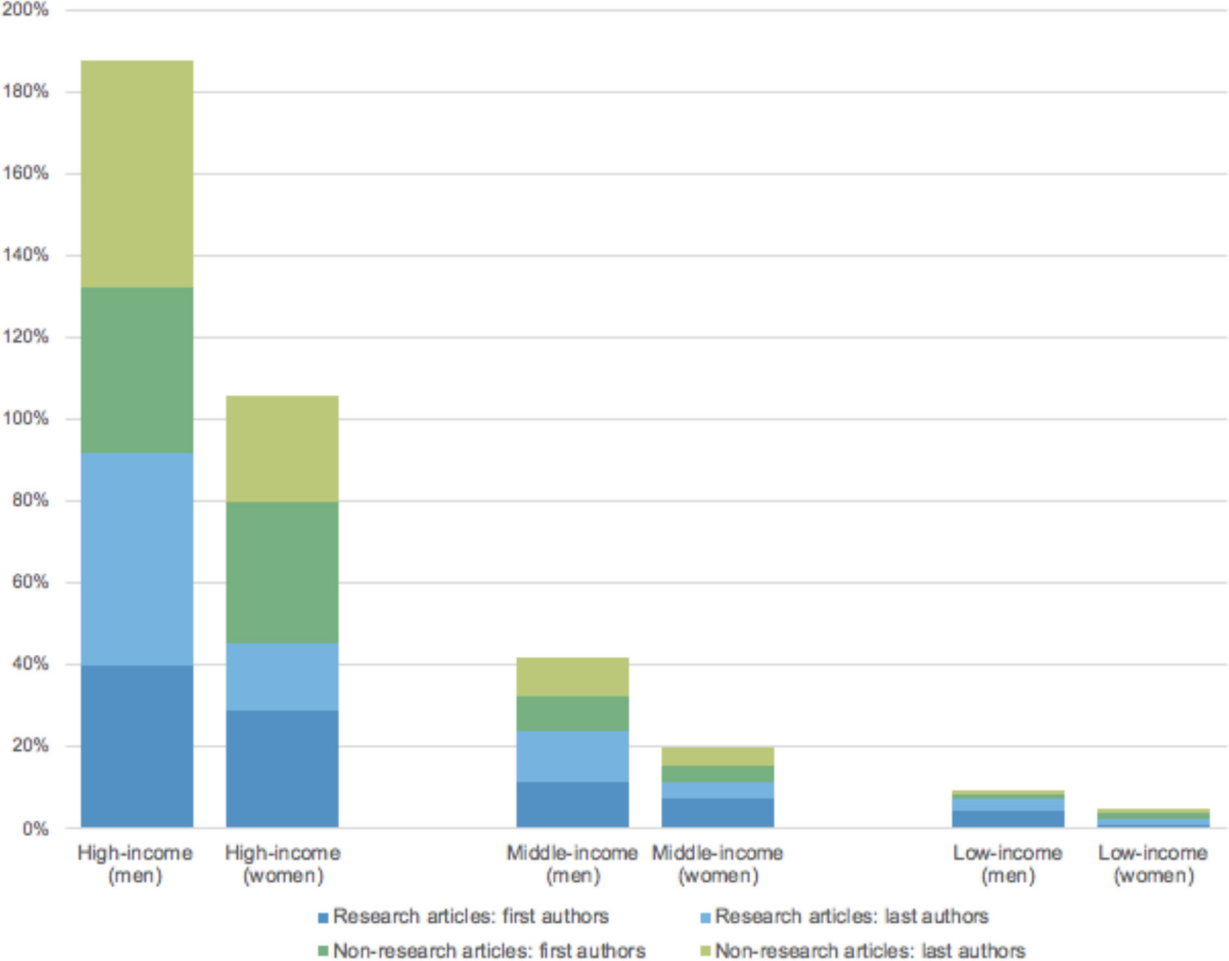
Percentage of first and last authors of research and non-research articles affiliated with high-, middle- and low-income countries, by gender.

In addition to gender, we collected information on the location of first and last authors’ institutional affiliation and classified these using World Bank criteria^34^ (table 3 and online supplemental table 3); country data for one author was not available. Authors affiliated with HICs accounted for 69% (194/280) of first and 74% (204/277) of last authors of research articles in our sample. These HIC authors accounted for 82% (206/250) of first and 82% (155/190) of last authors of non-research articles. Authors from middle-income countries represented 19% (54/280) of first and 16% (43/277) of last authors of research articles, and 13% (33/250) of first and 14% (26/190) of last authors of non-research articles. Authors from low-income countries represented 5% (30/557) of first and last authors of research articles, and 2% (9/440) of first and last authors of non-research articles in our sample (see figure 2). Authors affiliated with multiple institutions based in both high-income and low-income or middle-income countries accounted for 4% (42/997) of all authors.

10.1136/bmjgh-2021-005672.supp3 Supplementary data

The proportion of men first and last authors compared with women across income levels did not vary greatly: in HICs, we found 1.7 times as many men first and last authors as women; 2.1 times in middle-income countries, and 2.0 times in low-income countries. Across both research and non-research articles, women affiliated with institutions in low-income and middle-income countries represented 7% (36/518) of first authors and 5% (24/460) of last authors. Women affiliated with low-income countries represented 1% of last authors of both research and non-research articles in our study. However, we found that specialist global health journal articles consistently featured higher proportions of both men and women first and last authors from LMICs compared with the general medical journals (table 3).

**Figure 3:**
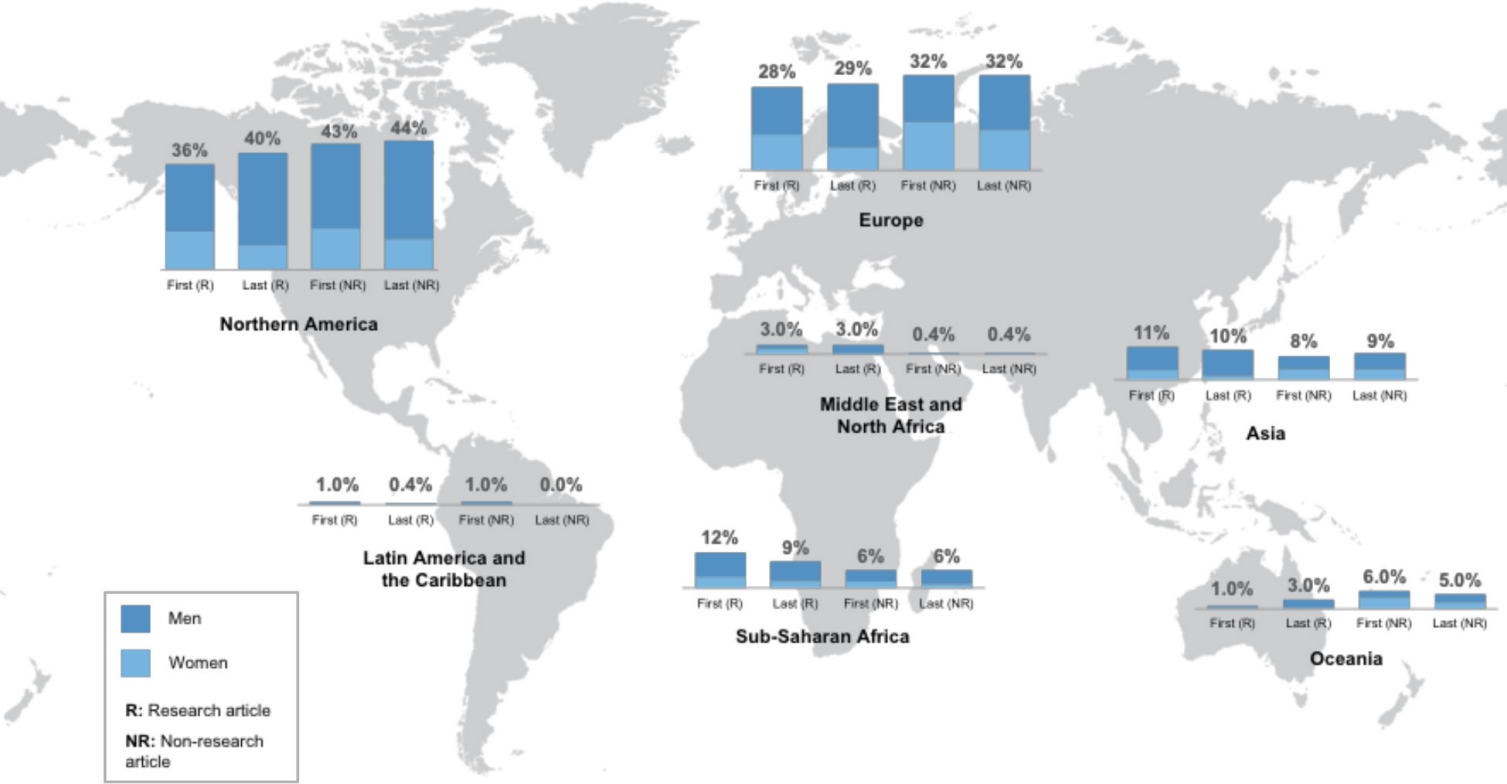
Regional distribution of institutional affiliation of authors: first and last authors of research articles, and first and last authors of non-research articles.

We also found disparities in the regional distribution of authorship (figure 3). Two-thirds (179/280; 64%) of first authors of research articles were affiliated with institutions in North America and Europe, and higher proportions of last authors of research articles, and first and last authors of non-research articles were affiliated with these two regions (69%, 75% and 76%, respectively).

Authors affiliated with institutions in Asia and sub-Saharan Africa accounted for between 9% and 12% of first and last authors of research articles, and 6%–9% of non-research articles. The number of first and last authors from the combined five regions of Asia, Latin America and the Caribbean, the Middle East and North Africa, Oceania and sub-Saharan Africa (n=242) was lower than the number of authors from either Europe (n=300) or North America (n=403).

#### Relationship between author characteristics and impact factor of journal

Women and authors affiliated with institutions located in LMICs were found to publish in journals with lower journal impact factors than men and authors affiliated with HICs. In our sample, articles led by female first authors were on average published in journals with impact factors 6.4 points lower than articles led by male first authors. Articles with female last authors in our study were published in journals with journal impact factors 11.7 points lower than male last authors. Articles with first and last authors from HICs were published in journals with journal impact factors nearly 20 points higher than articles with first authors from LMICs. All of these findings were statistically significant (table 5).

**Table 5:** Coefficients from linear regression of journal impact factor on research article author gender and affiliation

	Journal impact factor
**First author gender**	
Man	REF
Woman	−6.40*
**Last author gender**	
Man	REF
Woman	−11.71*
**First author affiliation**	
Low-income and middle-income countries	REF
High-income countries	19.18*
**Last author affiliation**	
Low-income and middle-income countries	REF
High-income countries	18.54*
**First author gender and affiliation**	
Women, low-income and middle-income countries	REF
Women, high-income countries	13.91*
Men, low-income and middle-income countries	1.92
Men, high-income countries	24.12*
**Last author gender and affiliation**	
Women, low-income and middle-income countries	REF
Women, high-income countries	7.06
Men, low-income and middle-income countries	0.01
Men, high-income countries	21.73*

*p<0.05.

In our sample, articles with male first and last authors from HICs were published in journals with journal impact factors more than 10 points higher than articles with female authors from HICs. This increased to more than 20 points when comparing male authors from HICs with either male authors from LMICs or with female authors from LMICs. Again, these findings were all statistically significant. However, we did not find a statistically significant difference in the journal impact factors of articles with women authors affiliated with LMICs as compared with men affiliated with LMICs (table 5).

Female first authors from HICs were published in journals with journal impact factors almost 14 points higher than articles with female first authors from LMICs. There was no statistically significant difference between the journal impact factors of female last authors from HICs and LMICs.

## Discussion

We found that published content within both general medical and specialist global health journals fails to consistently acknowledge and/or address dimensions of sex/gender in research. Additionally, we found a relationship between author characteristics and the likelihood that research incorporated sex and gender (women were more likely to incorporate sex and gender in their research), as well as the impact factor of the journals in which they publish (women publish in journals with a lower impact). Our findings build on previous work and demonstrate persistent inequalities.

Despite a variety of policies and guidance on incorporating sex and/or gender into research, publications remain largely sex and gender blind. Even when journals do include sex/gender reporting in their submission requirements, it frequently does not lead to any meaningful inclusion beyond the reporting of participants’ sex or gender. Among research articles, although sex and gender were frequently included in the analysis (59% of articles presented some form of sex/gender analysis), only a minority of authors explore the meaning of any observed differences in their discussion. These findings are similar to recent studies conducted^35 36^ and may even represent progress from previous studies looking at RCTs which found lower prevalence of sex/gender reporting.^37 38^

General medical journals were shown to perform better on a few key variables when compared with specialist global health journals, particularly at reporting participants’ sex or gender. However, global health journals were more likely to consider and include sex/gender in their discussion. These findings may be reflective of the different article types produced by the different journals, but emphasise the importance of consistent implementation of author guidelines covering sex/gender analysis from the start to the end of the article. With regards to qualitative studies, despite the acknowledgement of the gender dynamics between interviewer and interviewee and subsequent influence on data collected,^39^ these studies were significantly less likely to report sex/gender of participants or discuss the role and influence of gender in their research than the sample as a whole.

This study adds further evidence supporting demands for equitable and inclusive career structures within academic and research institutions. Findings suggest that not only are women less likely to be first or last authors, but even when they do occupy these lead positions, they are publishing in journals with lower impact factors than when compared with men first and last authors. The gender of first and last author takes on additional importance as women occupying lead authorship positions are more likely to publish research that takes sex and gender into account. These findings are in line with those of Sugimoto *et al*, who found in their analysis of over 11 million publications in the health and biomedical science fields, sex-related reporting was more common both in papers with female first and last authors, and those published in journals with lower journal impact factors.^16^ Additionally, our study demonstrated that for both research and non-research articles, women last authors tended to have more women listed as authors alongside them. This suggests that when women do ascend to lead research roles, they may play a role in expanding publication opportunities for other women.

Gender does not act alone to influence career progression. Our study took a multidimensional approach by examining the relationship between author gender, geographical location and publication characteristics, including the impact factor of the journal. Both women and men from LMICs are significantly underrepresented as first and last authors when compared with authors in HICs in our sample - with women affiliated with LMICs, in particular, found to be first authors on just 7% of articles and last authors on 5%. Similar findings have been noted in other studies.^24–26 40^ One additional finding in our work is that specialist global health journals have consistently higher proportions of both women authors and LMIC authors compared with general medical journals. Furthermore, among the articles included in our sample, LMIC authors were clustered in journals with lower impact factors which was particularly striking when compared with HIC authors who more often published in high impact journals. Again, this finding is consistent with studies addressing similar themes.^41 42^

### Strengths and weaknesses

This review focuses on sex/gender and equity in publishing. By considering multiple aspects of the publication process, including article guidelines, content as well as intersections between author characteristics, links between variables and areas of focus can be more easily identified.

The manual nature of data extraction allowed for this more in-depth analysis as well as a more accurate gendering of authors. Information was not based solely on gendering names as is often the method with other papers,^43^ but by gaining additional confirmation from pronoun usage in online bio sketch content. Manual data extraction, however, limited the number of articles that could be reviewed within the time-frame available, as compared with algorithmic studies.^16 44^ We acknowledge that the 20 research and 20 non-research articles included creates a snapshot, but may not be representative of all journals’ yearly outputs. Additionally, the extent to which articles performed a sex/gender analysis remained challenging to quantify and standardise especially when covering different research article types. Sex/gender analysis subsequently ranged from narrative comments to fully sex-disaggregated data. This likely led to an overestimate of articles meeting satisfactory sex/gender analysis in our findings compared with similar studies covering individual study types.^37^ Finally, this study acknowledges that sex and gender are but two of many intersecting determinants of health. The consideration of race, ethnicity and socioeconomic status, for example, is equally as important in exposing health inequalities and ensuring health research is robust, replicable and reproducible.

We acknowledge that a binary and cis-normative concept of gender was employed in our methodology. This reflects the way in which sex/gender is reported in vast majority of articles and not our understanding of gender as defined above. We recognise the broad spectrum of gender identities that are not defined by these categories, and the limitations this has for research involving sex and gender minorities.

Finally, it is important to recognise the way in which the author representation of our own publication contributes to the area of focus. The majority of our authors identify as women, and both men and women authors identify as feminists. Our research paper positively demonstrates collaboration and mentorship in action, with an emphasis on enhancing career opportunities for younger women. However, there remains less progress among geographical representation, with all our authors affiliated with HICs. While calling for journals to do better, it is vital research teams also consider their role in redressing global imbalances. This paper highlights the importance of creating partnerships between institutions and researchers in different regions of the world—as a positive step towards the equitable distribution of power and privilege in academic publishing. This is something GH5050 are working towards in other areas of our portfolio of activities.

### Implications of study

First, failing to appreciate sex and gender differences in medical and health research results in missed opportunities for identifying and responding to the impact that these factors have on health and well-being for all people.^45^ This is illustrated, for example, in the current COVID-19 pandemic. Men’s vulnerability to the virus is seen in their higher rates of severe illness and risk of death^46^—arising from the intersection of underlying biological risk combined with gendered determinants that appear to result in higher levels of concurrent diseases associated with risk of death. While women appear to have a lower risk of death, they nonetheless bear the brunt of the social, economic and other secondary impacts of the virus.^47^ However, much research is conducted without due consideration of sex and gender which undermines the development of tailored policy responses and programmatic and clinical interventions.

Second, failing to adequately represent authors affiliated with LMICs is particularly striking and problematic for the global health journals, which have traditionally focused much of their publishing output on health in LMICs.^48^ The lack of representation from populations that are the centre of much global health research limits knowledge production.^49^ Such under-representation arises from a range of factors, but needs acknowledging and addressing if global health research is to achieve a goal of being both equitable and effective.^50^

## Conclusion

General medical and specialist global health research fails to consistently apply a sex/gender lens and remains largely the preserve of (predominantly male) authors in HICs. The focus of our study on journals allows reflection on the process of publishing as it currently stands, while acknowledging that systemic change is required to advance towards both recognising the importance of gender and sex in health and well-being and promoting more inclusive gender and geographical representation within academic and research careers. Journals play a key role in shaping the narrative, setting priorities and enforcing existing guidelines. As such, they should be encouraged to enhance their advocacy for work from a more diverse and representative body of scientists and researchers, while working collaboratively with funding agencies, research commissioners, research agencies (eg, universities) and research users to foster and promote the importance of diversity in research and publishing.

## Data Availability

All data relevant to and included in the study are presented in the article or uploaded as supplemental information.
