# Supplementary material for: The gender and geography of publishing: a review of sex/gender reporting and author representation in leading general medical and global health journals
Source: BMJ Glob Health. 2021 May 13;6(5):e005672. doi: 10.1136/bmjgh-2021-005672 (PMC8118011; doi:10.1136/bmjgh-2021-005672)
Supplement: Supplementary data [file bmjgh-2021-005672supp001.pdf]

Supplementary Table 1.

Journal specifies sex/gender reporting in author guidelines+	Pre-specified sex/gender analysis	Reported gender of study participants	Stated how sex/gender was determined	Performed any form of sex/gender analysis	Sex or gender in discussion
NO	REF	REF	REF	REF	REF
YES	0.67	3.69*	0.56	1.67	0.73

* $p<0.05$
+ Journals that specify sex/gender reporting in the author guidelines before 2018 (the year in which the articles in this analysis were published) include JAMA, JAMA IM, Lancet, and Lancet GH.
