# Supplementary material for: The gender and geography of publishing: a review of sex/gender reporting and author representation in leading general medical and global health journals
Source: BMJ Glob Health. 2021 May 13;6(5):e005672. doi: 10.1136/bmjgh-2021-005672 (PMC8118011; doi:10.1136/bmjgh-2021-005672)
Supplement: Supplementary data [file bmjgh-2021-005672supp002.pdf]

Supplementary Table 2. Analysis by study type: proportion of RCTs and qualitative studies that incorporate sex/gender related reporting.

Sex/gender reporting framework	Total (N=280)	RCTs (N=44)	Qualitative studies (N=33)
Pre-specified sex/gender analysis	8%	0%	9%
Reported gender of study participants	72%	93%	55%
Percentage of female participants			
0%-25%	8%	13%	7%
25%-50%	43%	49%	14%
50%-75%	43%	38%	50%
75%-100%	7%	0%	29%
Reported participation of transgender/non-binary participants	0.7%	0%	0%
Stated how sex/gender was determined	6%	5%	6%
Performed any form of analysis of sex/gender differences (excluding 8 articles w/ single sex participants)	59%	66%	24%
Featured sex and/or gender in discussion	40%	27%	27%
