# Supplementary material for: The gender and geography of publishing: a review of sex/gender reporting and author representation in leading general medical and global health journals
Source: BMJ Glob Health. 2021 May 13;6(5):e005672. doi: 10.1136/bmjgh-2021-005672 (PMC8118011; doi:10.1136/bmjgh-2021-005672)
Supplement: Supplementary data [file bmjgh-2021-005672supp003.pdf]

Supplementary Table 3. Institutional affiliation of first and last authors by national income level and region, research and non-research articles

Income level and region - institutional affiliation	Research articles				Non-research articles			
	First authors		Last authors		First authors		Last authors	
	% first authors	% women	% last authors	% women	% first authors	% women	% last authors	% women
**High-income countries**	69% (194/280)	42% (80/191)	74% (204/277)	22% (44/204)	82% (206/250)	42% (87/206)	82% (155/190)	32% (50/155)
**Middle-income countries**	19% (54/280)	39% (19/49)	16% (43/277)	24% (10/41)	13% (33/250)	35% (11/31)	14% (26/190)	33% (8/24)
**Low-income countries**	5% (15/280)	15% (2/13)	5% (15/277)	33% (4/12)	2% (6/250)	67% (4/6)	2% (4/190)	50% (2/4)
**Dual affiliation**	6% (17/280)	35% (6/17)	5% (15/277)	33% (5/15)	2% (5/250)	60% (3/5)	3% (5/190)	20% (1/5)
**Northern America**	36% (102/280)	36% (37/102)	40% (110/277)	21% (23/110)	43% (108/250)	33% (36/108)	44% (83/190)	24% (20/83)
**Latin America and the Caribbean**	1% (3/280)	33% (1/3)	0.4% (1/277)	0% (0/1)	1% (2/250)	0% (0/2)	0% (0/190)	0% (0/0)
**Europe**	28% (77/280)	43% (33/77)	29% (81/277)	27% (22/81)	32% (81/250)	51% (41/81)	32% (61/190)	43% (26/61)
**Middle East and North Africa (MENA)**	3% (9/280)	71% (5/7)	3% (7/277)	0% (0/7)	0.4% (1/250)	0% (0/1)	1% (2/190)	0% (0/2)
**Sub-Saharan Africa (SSA)**	12% (33/280)	32% (10/31)	9% (26/277)	30% (7/23)	6% (16/250)	40% (6/15)	6% (12/190)	25% (3/12)
**Asia**	11% (32/280)	31% (8/26)	10% (27/277)	16% (4/25)	8% (20/250)	47% (9/19)	9% (17/190)	40% (6/15)
**Oceania**	1% (4/280)	50% (2/4)	3% (7/277)	14% (1/7)	6% (14/250)	64% (9/14)	5% (9/190)	44% (4/9)
**Dual affiliation**	7% (20/280)	55% (11/20)	7% (18/277)	33% (6/18)	3% (8/250)	50% (4/8)	3% (6/190)	33% (2/6)
**Total**	100%	40% (107/270)	100%	23% (63/272)	100%	40% (105/248)	100%	32% (61/188)

Notes: Single authors are included in the First Author calculation and excluded in Last Authors calculations. Where location of affiliation and/or gender of the author could not be determined, those authors were excluded from analysis, which accounts for shifting denominators. Eleven non-research articles authored by the journal were excluded from analysis.
